# Deep Learning Framework for Complex Disease Risk Prediction Using Genomic Variations

**DOI:** 10.3390/s23094439

**Published:** 2023-05-01

**Authors:** Hadeel Alzoubi, Raid Alzubi, Naeem Ramzan

**Affiliations:** 1 Department of Computer Science, College of Computer Science and Information Technology, King Faisal University, Al-Ahsa 31982, Saudi Arabia; 2 School of Computing, Engineering and Physical Sciences, University of the West of Scotland, High Street, Paisley PA1 2BE, UK

**Keywords:** complex diseases risk prediction, feature selection, GWAS, machine learning, mutual information, SNP

## Abstract

Genome-wide association studies have proven their ability to improve human health outcomes by identifying genotypes associated with phenotypes. Various works have attempted to predict the risk of diseases for individuals based on genotype data. This prediction can either be considered as an analysis model that can lead to a better understanding of gene functions that underlie human disease or as a black box in order to be used in decision support systems and in early disease detection. Deep learning techniques have gained more popularity recently. In this work, we propose a deep-learning framework for disease risk prediction. The proposed framework employs a multilayer perceptron (MLP) in order to predict individuals’ disease status. The proposed framework was applied to the Wellcome Trust Case-Control Consortium (WTCCC), the UK National Blood Service (NBS) Control Group, and the 1958 British Birth Cohort (58C) datasets. The performance comparison of the proposed framework showed that the proposed approach outperformed the other methods in predicting disease risk, achieving an area under the curve (AUC) up to 0.94.

## 1. Introduction

The human genome is the whole set of deoxyribonucleic acid (DNA) sequences in humans, which consists of approximately three billion base pairs [1,2]. Human genomes are almost identical; however, at least three million nucleotides per individual are different. The most common type of these genetic variations is Single Nucleotide Polymorphisms (SNPs). Studies have proven that SNPs are the most contributing markers in several complex and rare diseases [1]. Most SNPs are natural; however, certain SNPs are functional and affect the phenotype of interest, such as skin colour, height, infection, resistance, or responses to drugs.

Genome-wide association studies (GWASs) have proven their ability to unveil susceptibility variants in human diseases [3,4]. These studies provide a better understanding of diseases by enabling researchers to identify SNPs that significantly differ in frequencies between the affected and healthy individuals. GWASs have identified more than 4164 loci contributing to common complex diseases such as diabetes [5,6,7], cancer [8,9,10], and rheumatoid arthritis [11]. Moreover, GWASs allow researchers to develop models for complex disease risk prediction based on genetic information [12,13,14,15,16]. If the disease of interest can be identified at the early stage, specific therapy plans can be applied to delay or even prevent the outset of some diseases [17,18]. Building risk prediction models can contribute to personalised medicine becoming feasible by utilising an individual’s genome to predict disease risk and the response to treatment. However, the critical issue is how to predict disease risk accurately from a huge number of SNPs.

Many machine learning algorithms have been applied to SNP data analysis in order to build a model that is able to predict disease risk for unseen samples. However, the process of developing a risk prediction system using genomic data is computationally expensive due to the extremely high dimensionality number of features. Consequently, it has become essential to provide the classifier with a manageable number of discriminative SNPs. Moreover, selecting the most convenient classification algorithm for building the prediction model is also a crucial step for achieving high model performance. Traditional machine learning techniques have been successfully employed in GWASs for risk prediction [19,20]. However, deep learning networks are one of the most popular and powerful machine learning techniques for pattern recognition in many fields such as image processing [21], natural language processing [22], and bioinformatics [23,24,25]. Deep learning has the potential to learn high-level complex and hierarchical data patterns more accurately than traditional machine learning techniques. Multi-Layer Perceptron (MLP) is one of the state-of-the-art neural networks that consists of three main layers: input, hidden, and output. The hidden layers consist of two or more layers of self-learning neurons, where the weights of fully connected neurons between adjacent layers are learned using the backpropagation algorithm. Once the weights are set, samples from unseen targets can be used as input, allowing the framework to perform prediction. Convolutional neural networks (CNN) have been used to extract the most informative features to classify Alzheimer’s disease patients [26]. There have been some attempts to apply deep neural networks in the SNP dataset with promising performance [27,28,29].

Machine learning techniques have been effectively used in a variety of disease risk prediction systems based on human genomic variations [30]. The authors in [31] highlighted the recent development of machine learning algorithms in the field of genomic variations. They illustrated the contribution of machine learning techniques in improving complex disease prediction models. In [32], the authors built a model for assessing the risk of type 1 diabetes (T1D). Their model employed Support Vector Machines (SVM) as the classification algorithm, which was fed with 100 SNPs selected using an ensemble feature selection technique to achieve an AUC of 0.84. The authors in [33] developed a breast cancer risk prediction model using a combination of the MeanDiff feature selection technique and KNN classifier. Their proposed model showed a 10% increment in terms of accuracy over the baseline classifier; however, the best accuracy achieved by their system was less than 60%.

The authors in [34] compared five different machine learning methods, Bayesian Networks, SVM, Random Forest, Radial Basis Function network, and Logistic Regression, to predict the risk factors of bipolar disorder. The best performance was achieved using Bayesian Networks with an AUC of 0.556. In [35], the authors developed an ensemble machine-learning technique for autism disease risk prediction. In [36], the authors assessed risk prediction for complex diseases by comparing the performance of four different prediction algorithms. The best performance was reported using sparse penalised approaches. The authors in [37] applied different machine-learning techniques to predict the risk of anorexia nervosa. The logistic regression with the lasso penalty technique performed slightly better than SVM and gradient-boosted trees. They applied their system to different sample sizes of the dataset and concluded that a larger sample size improves the machine learning risk prediction outcomes. A similar conclusion was drawn by [15], who employed logistic regression over the top 3000 associated SNPs on the WTCCC [38] for Crohn’s disease. In this paper [39], the authors compared the ability of allele counting, Logistic Regression (LR), and SVM to predict coronary artery disease risk and found the best AUC up to 0.60 to be achieved by LR. In order to assess the risk of T1D and rheumatoid arthritis (RA), the authors in [40] employed SVMs and random forest. Their proposed system successfully reached an AUC of 0.82 and 0.71 for T1D and RA, respectively. The aforementioned works applied different machine learning techniques and reached promising performance in some cases. In order to predict breast cancer risk, the authors of [41] proposed a system that adopts a gradient tree boosting method followed by an adaptive iterative SNP search. The authors aimed to capture the group of interacting SNPs over the given disease. A combination of genomic data and demographic data has been used to predict the disease risk of breast cancer by [42]. Their system used a gradient tree-boosting method in both the selection and classification phases. However, many clinical and medical applications require more accurate prediction systems.

Risk prediction methods typically apply different techniques in order to select a manageable number of SNPs. Most studies rank SNPs based on the *p*-value of their association with the phenotype of interest to control the number of selected SNPs and use the top associated SNPs as input to a prediction algorithm [43]. However, the predictive power of these studies is relatively poor, and discarding SNPs with a low *p*-value could limit the opportunity to identify inter-SNP correlations [19,44]. Moreover, there are variations associated with many diseases that have not yet been identified; hereby, analysing an expanded list of SNPs may improve the prediction system performance. For example, in [19], it was suggested that considering uncommon and rare SNPs can improve risk prediction for some diseases such as Parkinson’s disease using SVM. In addition, SNPs were selected for SVM by applying different *p*-value thresholds. Moreover, in [44], the BootRank technique was used in order to select robust informative SNPs to be used in a risk prediction model. The BootRank technique was combined with seven different classifiers to evaluate the performance of their proposed technique. Their model improved the ability to predict the disease risk of unseen individuals in the WTCCC data.

In this work, an accurate deep-learning framework for complex disease risk prediction has been proposed. An adequate subset of SNPs that are highly correlated and non-redundant has been selected using the Joint Mutual Information (JMI) method [45]. Then, the selected features were fed to an MLP that consists of an input layer, five hidden layers, and an output layer to train the prediction system. The proposed system was evaluated using datasets from WTCCC. The comparative experimental results demonstrate the ability of the proposed to accurately predict risk for different diseases as compared to the state-of-the-art approaches including [15,32,34,39,40,44,46,47,48,49], achieving an AUC of up to 0.94. The rest of this work is organised into three sections. Section 2 discusses materials and methods. Section 3 presents the experimental results and discussion. Finally, Section 4 concludes this work.

## 2. Materials and Methods

### 2.1. Genotype Datasets

Genotype data were obtained from the WTCCC [38] for seven different diseases. The diseases are Type 1 diabetes (T1D), Type 2 diabetes (T2D), inflammatory bowel disease (IBD), coronary artery disease (CAD), bipolar disorder (BD), rheumatoid arthritis (RA), and hypertension (HT), as presented in Table 1. Each disease dataset contains approximately 2000 cases. The control sets obtained from the UK National Blood Service Control Group (NBS) and 1958 British Birth Cohort (58C) contained 1500 individuals [38]. Each sample consists of 500,568 SNPs that were produced by an Affymetrix 500k chip sequencer. As recommended by the associated datasets, 809 samples and 30,956 SNPs were excluded due to deviation from Hardy–Weinberg equilibrium, bad quality, or bad clustering [38]. The dataset has been filtered to exclude SNPs based on the following threshold: a Minor Allele Frequency (MAF) of 1%, *p*-value $<1\times 10^{-3}$, and a missing rate of 5% [32,46,50,51,52]. As a result of the filtering, the final number of samples for each data set is presented in Table 1, with 469,606 SNPs for each one. To ensure that our results are not biased to cases or control, an equal number of samples for each class have been used. Where a group of healthy samples were randomly selected from UKBS and 58C to keep the case:control ratio at 50%:50% for each disease.

Mutation exists in the gene copy that is inherited from both parents. The allele frequencies are represented by A and B for the major allele frequency and minor allele frequency, respectively. Any given SNP could have the value of AA or BB to indicate that it is a homozygous SNP and the value of AB for a heterozygous SNP. In this proposed work, we used the additive model to encode SNPs. The encoding technique counts the minor allele appearance. Consequently, the coding value 0 represents AA, 1 represents AB, and 2 represents BB. Finally, after implementing the aforementioned coding technique, the dataset is represented in numerical format.

If a dataset consists of *n* samples and *q* SNPs, which can be represented by a G=n×q matrix, then $G_{ij}$ is the number of the minor allele of SNP *j* for the sample *i*. Let $Y_{i}$ be a binary indicator for the disease status of a given sample i=1,⋯,n. The affected samples (case) are considered as having a positive class label ($Y_{i}=1$) and the healthy ones (control) as having a negative class label ($Y_{i}=0$).

### 2.2. Method

**Figure 1:**
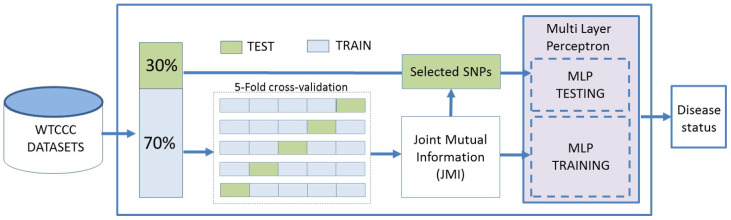
The proposed framework structure.

The proposed framework predicts the risk of an examined disease using SNP data. An MLP-based binary classifier has been developed to predict the disease risk status. A mutual information feature selection technique has been applied to decrease the feature space dimensionality and select the most discriminative SNPs. The dataset was split into (70%) training and (30%) testing sets, keeping the class ratio of each group similar to that of the whole dataset, and the testing data were only used for analysing the predictive power of the proposed system as illustrated in Figure 1. Five-fold cross-validation has been applied over the training data in order to perform feature selection. Finally, different performance metrics have been used to evaluate the predictive power of the proposed framework.

#### 2.2.1. Feature Selection

The extremely large number of SNPs in the genome makes the application of machine learning techniques on SNP data computationally impossible. Consequently, the application of feature selection techniques is necessary for the selection of a significantly smaller subset of SNPs. Statistical and machine learning-based feature selection methods have demonstrated their ability to select an optimal SNP subset out of the whole genome [35,53]. In this work, JMI has been employed as a feature selection method in order to reduce computational complexity and improve risk prediction performance.

Mutual information feature selection methods have been widely applied in the biomedical field [54]. Mutual information is used to measure the features’ relevancy and redundancy [55]. In the multivariate filter selection method, mutual information does not make any assumption of the data or change the original data representation [56]. In this work, JMI was used to measure the discriminative power of features and select a reduced set of SNPs to be injected into the prediction model.

JMI is a popular feature selection technique that selects a subset of features to maintain a high feature association and maximum correlation with the class of interest [45]. This method measures the information provided by the feature vector $s_{1},s_{2},\cdots ,s_{q}$ that decreases the uncertainty about the class label *Y*. JMI uses mutual Information to measure the amount of relevancy and redundancy between features. JMI calculates not only mutual information between features and the class label but also takes into consideration the correlation between the new feature and already selected features *D*, thus ensuring a good trade-off between relevancy and redundancy [45]. A higher JMI value for a feature $s_{i}$ means that the feature $s_{i}$ is relevant to the target *Y* and is highly complementary to the already picked features $s_{j}$, *j* in *D*. The JMI for a feature $s_{q}$ is computed as shown in Equation (1) [57].

(1) $$JMI\left(s_{i}\right)=\sum_{s_{j}\in D}I\left(s_{i},s_{j};Y\right)$$

where:

(2) I(X,Y;Z)=I(X;Z|Y)+I(Y;Z)

(3) $$I\left(Y;Z\right)=\sum_{y,z}p\left(y,z\right)\log\frac{p(y,z)}{p(y)p(z)}$$

(4) $$I\left(X;Y|Z\right)=\sum_{y\in Y}p\left(y\right)\sum_{x\in X}\sum_{z\in Z}p\left(xz|y\right)log\frac{p(xz|y)}{p(x|y)p(z|y)}$$

In this work, JMI was applied over the training set to select a subset of SNPs, *D*. *F*-fold cross-validation has been applied in order to create a matrix q×F, with *q* being the number of SNPs of each sample and F=5. At any given fold, the subset of selected SNPs is assigned to 1, and the value of 0 is assigned to the remaining unselected ones as presented in Equation (5).

(5) $$d_{j}=\left\{\begin{array}{cc} 0 & \mathrm{if}\;\mathrm{SNP}j\;\mathrm{is}\;\mathrm{not}\;\mathrm{selected}\\ 1 & \mathrm{if}\;\mathrm{SNP}j\;\mathrm{is}\;\mathrm{selected} \end{array}\right.$$

**Figure 2:**
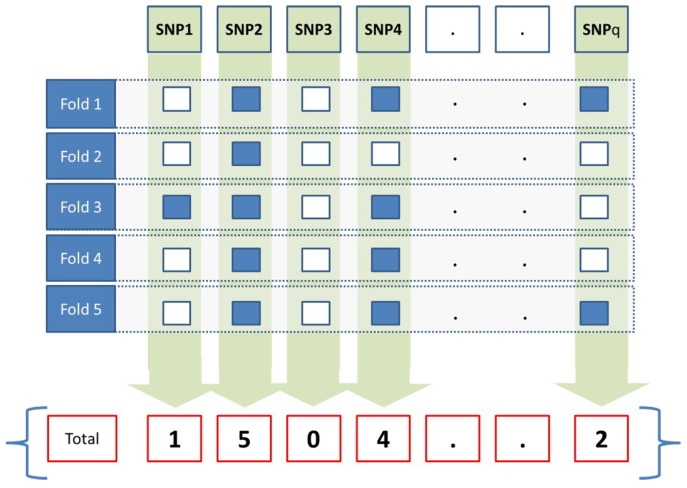
The feature selection method.

At the end of the fifth fold, the accumulated weight *W* for a given SNP *j* is calculated as presented in Equation (6). The weight for each SNP represents how many times a given SNP was selected, as illustrated in Figure 2. For example, if an SNP weight is 1, that means it has been selected in all folds. An SNP weight will be 0.6 if it has been selected in three folds. An SNP weight will be 0 if it has not been selected in any fold. Only SNPs that have a weight exceeding a threshold value (a) will be propagated to the prediction model.

(6) $$W_{j}=\frac{\sum_{t=1}^{5}d_{j}}{5}$$

#### 2.2.2. Deep Learning

**Figure 3:**
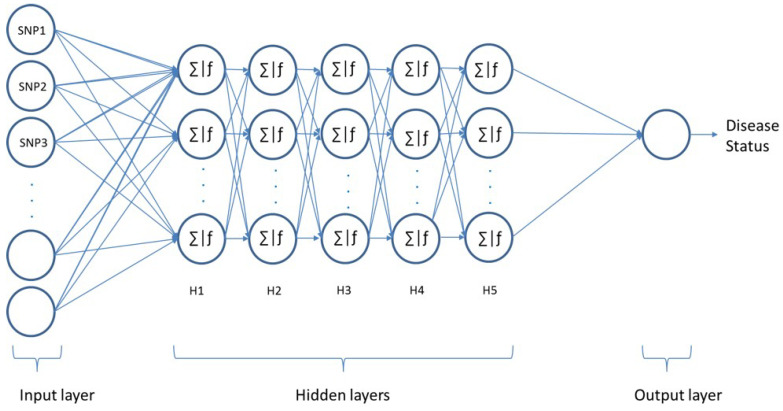
The multilayer perceptron framework.

Artificial neural networks (ANNs) are modelling tools inspired by the function of neurons in the human brain. These networks offer an alternative way to handle complex problems and are able to perform predictions for linear/nonlinear problems. Multi-Layer Perceptron (MLP) is one of the popular feed-forward neural networks that consists of an input, hidden, and output layer. In this work, the feedforward MLP consisting of one input layer and one output layer along with five hidden layers has been employed, as conceptualised in Figure 3. Each layer contains a number of neurons, which are interconnected in multiple layers by weighted connections.

The input feature vectors are passed through the multiple hidden layers downstream to the output layer [58]. The feature vectors are combined with weights to identify the informativeness of the inputs to the next layer. For any given neuron in layer *L*, the input is the sum of the weights for each neuron with a bias after applying an activation function in layer L−1.

Given data input $x_{i}\left(i=1,2,3,\ldots ,N\right)$, the neural model output *y* can be gained by Equation (7)

(7) $$y=f(\sum_{i=1}^{N}W_{i}x_{i}+b_{i})$$

where *W* is the model weight, *b* is the bias vector, and *f* is the activation function.

In this work, an MLP has been employed in order to identify patients with a certain disease, as conceptualised in Figure 3. The input layer consists of *N* nodes and considers SNPs as features. The output layer consists of one neuron (affected or healthy). The proposed models’ hyperparameters have been optimised using a grid search of the k-fold cross-validation technique with *k* = 3. This technique can ensure how accurately the model would perform in practice and avoid overfitting. Since we are implementing our model on different diseases, a modular model consisting of multiple modules is used. While similarities between models are possible, training the models separately means that all the architectures are optimised using various hyperparameters. After implementing a grid-based search, all possible hyperparameter value combinations have been examined. The best performances have been achieved with five hidden layers and 512 neurons in each hidden layer for all dataset models. Different activation functions for the hidden layers were evaluated: the tanh function performed the best out of the examined activation functions in three datasets, namely RA, T1D, and T2D, while the relu function performed better in CAD and IBD. The best performance of the HT dataset has been achieved using the sigmoid function. The softmax function was used in the output layer for all models. The softmax activation function used in our model is presented in Equation (8).

(8) $$softmax\left(x\right)=\frac{e^{\left(x_{i}\right)}}{\sum_{j=1}^{k}e^{\left(x_{j}\right)}}$$

where *x* is the input vector to the softmax function, $x_{i}$ is the *i*th element of the input vector, and *k* is the number of classes.

Different optimizers were used as a learning algorithm. The Adam optimizer outperforms the compared optimisation functions in most models. For BD and HT datasets, the best optimizers were NADAM and rmsprop activation functions, respectively. However, for the other parameters, all models achieved their best performance using the same values. In order to avoid model overfitting, a dropout technique that drops neurons randomly along with their connections has been used with a probability of 0.6. The best performance was achieved using 200 epochs for all models. Finally, the best learning rate was achieved using a 0.001 learning rate. The proposed models can be validated using test data in order to demonstrate their high-performance ability. Possible hyperparameter values are given in Table 2.

After building the networks and optimising the parameters on the seven aforementioned complex disease datasets, we came up with more than one model: the first one uses the same hyperparameters for three datasets (RA, T1D, and T2D), and the model uses the Adam optimizer and tanh activation function. A slight difference was implemented by using the relu activation function in the CAD and T1D datasets. On the other hand, two more models were implemented using NADAM, tanh and rmsprop, sigmoid as optimiser and activation functions, respectively. Finally, the proposed models can be validated using test data in order to demonstrate their high-performance ability.

The cross-entropy cost function, which is explained in Equation (9), has been used to estimate the output error.

(9) $$Loss=-\frac{1}{N}\sum_{i=1}^{N}\left[t_{i}\log\left(p_{i}\right)+\left(1-t_{i}\right)\log\left(1-p_{i}\right)\right]$$

for *N* data points, where $t_{i}$ is the truth value taking a value 0 or 1, and $p_{i}$ is the Softmax probability for the *i*th data point.

#### 2.2.3. Evaluation

Different experiments were conducted to evaluate the performance of the examined deep learning prediction architecture in terms of accuracy (Equation (10)), sensitivity (Equation (11)), precision (Equation (12)), F1-score (Equation (13)), AUC, and Matthews correlation coefficient (MCC) (Equation (14)). In order to compute the metrics, different values were calculated: 1. True positive (TP): the number of samples that were correctly identified to be corresponding to the targeted disease. 2. False positive (FP): the number of samples that were wrongly identified to correspond to the targeted disease. 3. False negative (FN): the number of samples that were wrongly classified as healthy. 4. True negative (TN): the number of samples that were correctly classified as healthy.

(10) $$Accuracy=\frac{TP+TN}{TP+FN+FP+TN}$$

(11) $$Sensitivity=\frac{TP}{TP+FN}$$

(12) $$Precision=\frac{TP}{TP+FP}$$

(13) $$F1\;\;score=2\cdot \frac{Precision\cdot sensitivity}{Precision+sensitivity}$$

(14) $$MCC=\frac{\left(TP*TN-FP*FN\right)}{\sqrt{\left(TP+FP)(TP+FN)(TN+FP)(TN+FN\right)}}$$

Each dataset was split into two main parts: 70% for training the system and 30% for testing the system. Furthermore, in order to identify the optimum features subset, a five-fold cross-validation technique was implemented to train data during the feature selection process. To this end, the training samples were shuffled and split into five groups. The splitting kept the ratio of the classes similar in each group to that of the original dataset. Then, the experiment of selecting features was repeated five times, and at each fold we used one group for testing the model and the remaining four groups as a training set. The final classification performance results were computed using the 30% of the original dataset that was unseen by the feature selection and training phases.

At the end of the fifth fold of the feature selection process, each SNP had a weight value, depending on how many times the SNP has been selected. SNPs with weights larger than the threshold value *a* were selected for the final feature vector.

## 3. Results and Discussion

**Figure 4:**
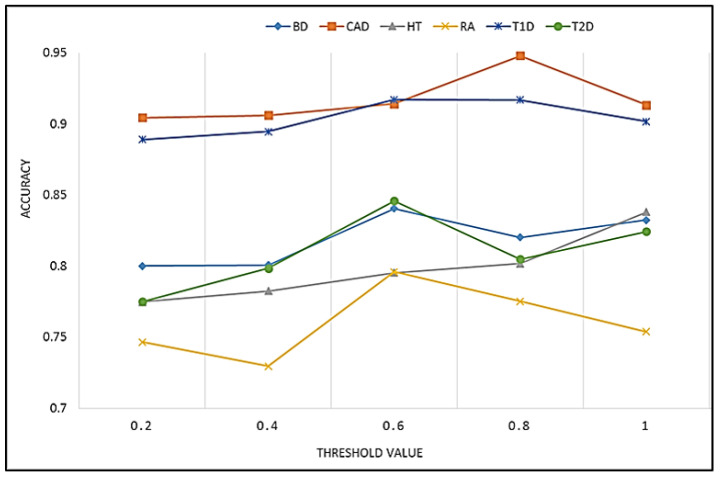
The performance evaluation using different thresholds over seven datasets.

The threshold value was selected experimentally, by evaluating different values and selecting the optimal value, as shown in Figure 4 and Table 3. In most datasets, the best prediction accuracy was achieved using a threshold of 0.6. However, in predicting the risk of CAD and HT, the best performance was achieved using a threshold value of 0.8 and 1, respectively.

The performance of the examined prediction deep neural network approach for each dataset in terms of accuracy, sensitivity, precision, F1 score, and MCC is presented in Table 4. The achieved results demonstrate the ability of the proposed disease risk prediction system to perform accurately. The affected samples were identified with an accuracy range between 0.796 to 0.948 for seven different complex disease datasets. Regarding the sensitivity and precision values, the proposed system was able to detect most patients in the datasets with high sensitivity values in most cases, ranging from 0.798 for HT disease to 0.934 for T1D disease. Moreover, very few healthy samples were identified as a case with precision values ranging from 0.83 to 0.966 for all diseases, apart from IBD disease, with a precision of 0.726. The proposed system performed the best in identifying the risk of CAD with an F1 score reaching 0.95. In predicting the risk of T1D, T2D, BD, HT, and RA, the F1 score of the proposed system ranged between 0.84 and 0.92. However, predicting the risk of IBD disease was the most challenging, achieving an F1 score of up to 0.782. For the MCC, the performance of our proposed system ranged between 0.606 and 0.901.

**Figure 5:**
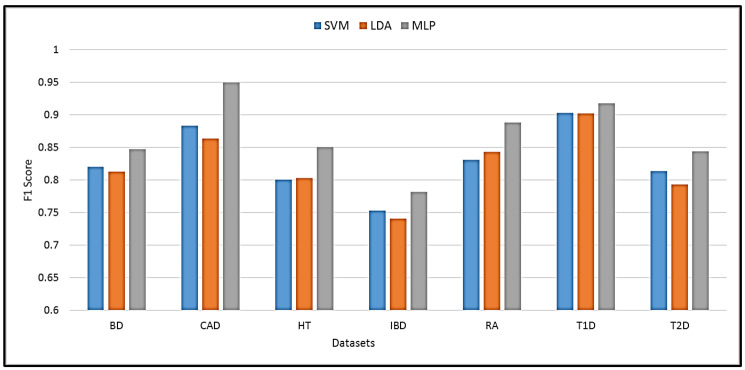
Performance comparison of the proposed method against SVM and LDA classifiers.

The proposed MLP was compared with other state-of-the-art machine learning techniques. The best performance with the compared techniques was achieved using SVM and linear discriminant analysis (LDA). A comparison of SVM, LDA, and the proposed MLP performance in terms of F1 score is shown in Figure 5. It is evident that MLP achieves the highest prediction performance with an improvement of 1.2% to 7.9% over SVM and LDA. The best improvement was achieved in the HT dataset, while the lowest was obtained in the T1D dataset.

Finally, we compared our proposed system prediction performance against other studies conducted on the WTCCC datasets as presented in Table 5. Comparing with these studies, we can guarantee that their systems dealt with datasets that have the same properties, the same number of controls and cases, and the same genotyping density. The AUC of the compared methods varied between 0.56 and 0.90 depending on the dataset and the algorithm. The proposed system outperformed the other frameworks for all datasets. The improvement of the proposed system in terms of AUC was less than 4% in identifying affected samples of T1D, T2D, and BD datasets. A better improvement of approximately 9% was achieved in predicting the risk of IBD. The risk prediction for RA, CAD, and HT was the best with an improvement of more than 15% over the best competitors.

Applying deep learning techniques to complex disease genomic datasets is not a trivial task, and the pre-processing of the data can be highly affected by many factors leading to a severe impact on the final conclusions. The proposed framework was able to select a subset of high discriminative SNPs that contributed to improving the prediction ability. A number of SNPs that have been identified with high discriminative values in our proposed system have been previously identified to be associated with diseases in other published works. Out of the selected SNPs, 75 SNPs were identified to be highly correlated with different diseases in the original dataset [38], 23 SNPs were identified in a study conducted over the same dataset [59], and 9 SNPs were identified in a study conducted only on the HT dataset [60].

## 4. Conclusions

In this work, a deep learning approach using MLP has been proposed to predict the risk of complex diseases based on genomic variations. The proposed approach exploits the JMI filter feature selection method in order to select a subset of SNPs with high discriminative power. The selected features are then fed to an MLP-based prediction algorithm to distinguish between healthy and affected samples. The proposed model has been evaluated on seven state-of-the-art datasets from WTCCC, UKNBS, and 58C. The experiment results demonstrate the superiority of the proposed model as compared to the traditional machine learning techniques, achieving an F1-score of 0.94. Moreover, the obtained results have been compared with state-of-the-art methods that were applied on the same datasets. An improvement in terms of an AUC of up to 22% compared to previous methods was achieved using the proposed approach. The proposed framework was also able to identify a number of SNPs that have high discriminative value and were previously identified to be linked with diseases in other published work. Taking into consideration the obtained prediction performance, as well as the performance of other methods proposed in the literature, it is evident that the proposed approach is applicable and efficient for complex disease risk prediction from SNP data.

## Figures and Tables

**Table 1:** Case and control datasets used.

Dataset	No. of Samples	No. of Excluded Samples	No. of Samples after Filtration
Bipolar disorder (BD)	1998	129	1869
Coronary artery disease (CAD)	1998	62	1936
Inflammatory bowel disease (IBD)	2005	256	1749
Hypertension (HT)	2001	48	1953
Rheumatoid arthritis (RA)	1999	136	1863
Type 1 diabetes (T1D)	2000	37	1963
Type 2 diabetes (T2D)	1999	75	1924
UK National Blood Service (UKBS)	1500	42	1458
1958 British Birth Cohort (58C )	1504	24	1480

**Table 2:** Hyperparameters and their range for MLP models.

Hyperparameter	Description	Range
Activation function	Neuron’s activation function	Relu, Sigmoid, tanh
Optimizer	The optimisation algorithm that performs the learning process in a neural network	rmsprop, NADAM, ADAM, SGD
Epochs	Number of learning iterations	50, 100, 200, 300
Learning Rate	Weight change updated during learning	0.001, 0.0001, 0.00001
No. of hidden nodes	No. of neurons in the hidden layer	64, 128, 256, 512
Dropout	Dropping out nodes during training	0.2, 0.4, 0.6
Mini batch size	Group size submitted to model during training	16, 32, 64, 100

**Table 3:** The number of selected features at each threshold value for all datasets.

Fold	BD	CAD	HT	IBD	RA	T1D	T2D
0.2	1991	2053	1767	1988	1758	1603	2224
0.4	1167	1099	1147	1183	1128	1121	1121
0.6	830	794	878	832	882	907	750
0.8	602	607	695	597	705	764	555
1	410	447	513	400	527	605	350

**Table 4:** Prediction performance for complex diseases.

	Accuracy	Sensitivity	Precision	F1-Score	MCC
BD	0.839	0.812	0.882	0.846	0.697
CAD	0.948	0.934	0.966	0.950	0.891
HT	0.838	0.798	0.904	0.848	0.685
IBD	0.796	0.847	0.726	0.782	0.606
RA	0.885	0.884	0.886	0.885	0.764
T1D	0.917	0.901	0.936	0.918	0.901
T2D	0.846	0.857	0.831	0.844	0.696

**Table 5:** Performancecomparison of the proposed prediction system and studies conducted on the WTCCC dataset in terms of AUC.

Disease/Method	T1D	T2D	BD	IBD	CAD	RA	HT
Proposed Model	0.92	0.85	0.84	0.79	0.94	0.89	0.84
BootRank [44]	0.90	0.82	0.83	0.70	0.72	0.74	0.68
GWASRank [44]	0.88	0.69	0.68	0.67	0.72	0.75	0.65
LO, AC [46]	0.75	0.60	0.67	0.63	0.60	0.67	0.61
DeepCOMBI [49]	0.65	0.65	0.65	0.65	0.65	0.65	0.65
SVM [40]	0.82	-	-	-	-	0.71	-
GWASelect [47]	0.79	-	-	-	-	-	-
SVM, LR [32]	0.89	-	-	-	-	-	-
Forward ROC [48]	-	-	-	-	-	0.71	-
LR, SVM, RF, BN [34]	-	-	0.56	-	-	-	-
Elastic-net [15]	-	-	-	0.64	-	-	-
LR, AC, SVM [39]	-	-	-	-	0.60	-	-

## Data Availability

The source datasets are publicly available from their respective sources.
